# Detecting sleep deprivation from running biomechanics using machine learning classification: a comparison between wearable and laboratory motion capture

**DOI:** 10.3389/fbioe.2026.1939545

**Published:** 2026-10-30

**Authors:** Maura Seynaeve, Kilian Hendrickx, Benedicte Vanwanseele, Toon de Beukelaar

**Affiliations:** 1 Department of Movement Sciences, Neuroscience of Movement and Performance Research Group, KU Leuven, Leuven, Belgium; 2 KU Leuven Institute of Sports Science, Leuven, Belgium; 3 Department of Computer Science, KU Leuven, Leuven, Belgium; 4 Department of Movement Sciences, Human Movement Biomechanics Research Group, KU Leuven, Leuven, Belgium

**Keywords:** biomechanics, fatigue, machine learning classification, recreational runners, running gait, sleep deprivation, wearable sensors

## Abstract

Sleep deprivation is associated with impaired endurance performance and an increased risk of running-related injury. Previous research has identified alterations in running biomechanics following a single night of sleep deprivation under laboratory conditions. However, whether these biomechanical changes can be detected using wearable technology remains unknown. Twenty-one recreationally active runners completed submaximal treadmill running under both normal sleep and total sleep deprivation conditions in a randomized crossover design. Biomechanical features were extracted simultaneously using a full-body motion capture system and a trunk-mounted wearable sensor. Five machine learning classifiers were evaluated in two classification tasks: a within-subject task using paired recordings from the same individual, and a between-subject task performed without individual baseline data. Within-subject classification exceeded chance level (label-permutation testing, p<0.05 ) for most models and both measurement systems, with best accuracies of 84% for the wearable sensor (Logistic Regression) and 81% for the motion capture system (Random Forest). These findings indicate that sleep deprivation produces a systematic and individually consistent biomechanical signature during running. In contrast, between-subject classification failed across nearly all models and systems, with accuracies remaining close to chance level (˜50%), suggesting that inter-individual variability obscures the sleep-deprivation signal in the absence of personalized baseline data. Both systems converged on temporal organization, loading-related variables, and stride-to-stride variability as the most discriminative feature domains. Contrary to expectations, the laboratory motion capture system did not outperform the wearable sensor. Together, these findings suggest that individualized, baseline-referenced monitoring may be important for detecting sleep-deprivation-related changes in running gait, and suggest that a single trunk-mounted wearable sensor may provide a practical solution for real-world monitoring when paired recordings are available.

## 1 Introduction

Recreational running is one of the most popular forms of physical activity worldwide, with millions of people participating regularly across all ages and fitness levels (Hulteen et al., 2017). While running offers well-established health benefits (Chul et al., 2014), it carries a substantial injury burden. Previous research suggests that as many as 65% of runners experience at least one overuse injury each year, with common examples including patellofemoral pain syndrome, iliotibial band friction syndrome, and medial tibial stress syndrome (Lopes et al., 2012; Messier et al., 2018). These injuries arise from the repetitive mechanical loading inherent to running, where cumulative stress applied to muscles, tendons, and joints may eventually exceed a structure’s tolerance threshold (Bertelsen et al., 2017). Importantly, fatigue is a key modulator of this process: as fatigue develops, running biomechanics change in ways that can introduce atypical loading patterns, potentially pushing tissues beyond their limits (Zandbergen et al., 2023). Because these biomechanical alterations are often subtle, objective monitoring tools are essential for coaches and clinicians working with this population (Mason et al., 2022).

When assessing exercise-induced fatigue, laboratory-based techniques have frequently been used; however, wearable sensors have more recently emerged as a practical tool for detecting such fatigue, defined as the acute, task-related decline in performance during or following a sustained effort (Marotta et al., 2022). Several studies using trunk- or shank-mounted accelerometers have characterized how impact-related and temporal gait parameters change with fatigue during running. Using a single trunk-mounted accelerometer during a treadmill run to volitional exhaustion, Schütte et al. found that fatigue increased horizontal-plane (mediolateral and anteroposterior) trunk-acceleration variability and reduced the regularity and predictability (sample entropy) of anteroposterior acceleration patterns (Schütte et al., 2015). Using continuous inertial sensor monitoring across an entire marathon, Reenalda et al. similarly found increased peak center-of-mass acceleration and decreased peak knee flexion by the end of the race relative to the start in three runners, alongside individually variable changes in stride length and step frequency (Reenalda et al., 2016); more broadly, peak tibial and sacral acceleration have been shown to increase with fatigue in running, suggesting greater impact loading as fatigue develops (Reenalda et al., 2019). Changes in shock attenuation between body segments have also been reported, with several studies observing increased head-to-tibia and sacrum-to-tibia attenuation under fatigued conditions, indicating altered impact transmission strategies (Derrick et al., 2002; Encarnación-M et al., 2023). Fewer studies have reported fatigue-related changes in spatiotemporal gait parameters such as stride length, stride frequency, and contact time, and these findings are less consistent; for instance, Meyer et al., using foot-worn inertial sensors across a marathon, observed gradual, progressive changes in contact time, flight time, stride length, speed, and leg stiffness over the course of the race, with two additional abrupt changes (“hitting two walls”) superimposed around 25 and 35 km (Meyer et al., 2021). Collectively, this body of work demonstrates that wearable accelerometers are sensitive to fatigue-induced biomechanical shifts during running.

However, fatigue is a multifactorial phenomenon that can arise from a variety of physiological and psychological stressors. Alongside exercise-induced fatigue, which arises from the accumulation of physiological and neuromuscular stress during prolonged or intense exercise, insufficient sleep represents a clinically important and underexplored source of fatigue for athletic populations (Charest and Grandner, 2022). Survey data using validated instruments such as the Pittsburgh Sleep Quality Index indicate that just over half of elite athletes across a range of Olympic sports screen positive for poor sleep quality (Halson et al., 2022). Among recreational runners specifically, a latent-profile analysis of 425 runners classified 37% as “Poor Sleepers,” a profile characterized by short sleep duration, low sleep quality, and frequent sleep problems (de Jonge and Taris, 2025). Beyond its prevalence, sleep deprivation also carries a measurable performance cost. A recent meta-analysis of 31 randomized and non-randomized controlled trials compared total or partial sleep deprivation with habitual sleep. Sleep deprivation had a moderate negative effect on endurance performance overall, and this effect was larger for longer-duration exercise (>30 min) than for shorter exercise (Lopes et al., 2023). In addition, a 26-week prospective cohort study of 339 runners found that poorer sleep quality was associated with a 36% higher risk of running-related injury (Goldberg et al., 2025). Despite these effects on running-related injury, studies examining the direct biomechanical consequences of fatigue induced by sleep deprivation remain scarce.

The biomechanical consequences of fatigue induced by sleep loss cannot be assumed equivalent to the consequences of exercise-induced fatigue as described above. Unlike the peripheral muscular fatigue caused by metabolite accumulation or energy substrate depletion, sleep deprivation is more likely to influence motor performance through central mechanisms, occurring proximal to the neuromuscular junction. Beyond its well-documented effects on cognitive performance (Killgore, 2010), sleep loss has been shown to impair motor performance in a task-dependent manner (Fullagar et al., 2015; Knowles et al., 2018; Vitale et al., 2019; Walsh et al., 2021; Craven et al., 2022). In general, effects are more pronounced for continuous, multi-joint movements (e.g., walking, running, or cycling) than for discrete, single-joint tasks (e.g., isolated knee-extension strength tests) (Fullagar et al., 2015; Knowles et al., 2018; Vitale et al., 2019; Walsh et al., 2021; Craven et al., 2022). This distinction is mechanistically meaningful: complex, continuous movements such as maintaining balance or walking rely on higher-order neural control processes, including feedforward planning, sensorimotor feedback, and executive attention, all of which are sensitive to sleep loss (Woollacott and Shumway-Cook, 2002; Pruszynski et al., 2011; Stuhr et al., 2018; Umemura et al., 2022). Running, as a dynamic, rhythmically coordinated, multi-joint task, is therefore likely to be vulnerable to these centrally mediated disruptions. Supporting this, recent work using a marker-based 3D motion capture system and instrumented treadmill confirmed meaningful biomechanical changes following a single night of sleep deprivation, including a lower cadence, longer ground contact time, greater knee flexion (a “Groucho”-like pattern), a distal-to-proximal shift in joint power, and reduced leg stiffness (Seynaeve et al., 2026). However, such laboratory-grade equipment is rarely accessible outside of research settings. Hence, this raises the question whether these alterations could instead be detected using wearable technology, a more scalable and ecologically valid alternative.

A frequent challenge in detecting these condition-induced biomechanical patterns is that inter-individual differences in gait patterns routinely exceed the magnitude of condition effects, causing group-level analyses to wash out the very signal of interest. As has been demonstrated in the exercise-induced fatigue literature, the average runner does not resemble any individual in the group, and population-level models frequently mask meaningful individual responses (Chalitsios et al., 2024). Machine learning has emerged as a promising framework for addressing this problem, precisely because it captures coordinated patterns across many biomechanical variables simultaneously rather than testing each in isolation. Early research established the feasibility of this approach for exercise-induced fatigue detection. For instance, Buckley et al. demonstrated that a single inertial measurement unit (IMU) placed at the lumbar spine could classify non-fatigued versus fatigued states using a single group-level model with an accuracy of 75% (Buckley et al., 2017). Additional work showed that this could be improved substantially through individualization. Subject-specific models consistently and significantly outperform group-based equivalents in identifying biomechanical shifts (Buckley et al., 2017; Op De Beéck et al., 2018; Dimmick et al., 2023). This performance gap exists because the most discriminative gait variables are highly subject-dependent; for instance, the specific mechanical features that signal fatigue or environmental adaptation in one runner often hold little predictive power for another (Marotta et al., 2021). To reliably isolate these subtle shifts, successful models typically require normalizing feature values against an individual’s unique baseline rather than using absolute population standards (Op De Beéck et al., 2018). This preprocessing step is essential to distinguish true condition-induced signals from a runner’s inherent movement style.

In conclusion, sleep deprivation represents a clinically relevant stressor for running populations, with established consequences for both performance and injury risk. Yet, despite the availability of both wearable technology and machine learning frameworks capable of detecting fatigue-related gait alterations, no study has examined whether sleep-deprivation-induced biomechanical changes during running are detectable using these tools. Therefore, the primary aim of this study was to determine whether sleep deprivation can be detected from running biomechanics using data from a single trunk-mounted inertial measurement unit (IMU). In addition, we will (i) compare the discriminative performance of a laboratory-based motion capture system (Vicon) and a wearable inertial sensor (Runeasi) in capturing sleep-deprivation-induced gait alterations, and (ii) evaluate the relative effectiveness of within-subject, paired classification and population-level classification approaches. Both modeling strategies were applied across motion capture systems, enabling a direct comparison between personalized models that leverage subject-specific baselines and generalized models that operate across individuals. We hypothesized that sleep deprivation would be detectable above chance level, with superior performance observed for within-subject classification compared to population-level models due to reduced inter-individual variability. Furthermore, although both motion capture systems were expected to demonstrate discriminative capacity, we anticipated that the laboratory-based motion capture system would outperform wearable inertial sensor owing to its richer and higher-resolution biomechanical feature space.

## 2 Materials and methods

### 2.1 Participants

A total of 22 healthy, recreationally active runners (18–35 years) were initially recruited. Eligible participants were required to have adequate baseline sleep quality and duration (Pittsburgh Sleep Quality Index score ≤ 5, Epworth Sleepiness Scale score <11, and self-reported habitual sleep duration >7 h/night), regular running habits ( ≥ 20 km/week for the past 6 months), and no running-related injuries in the preceding 2 months. Individuals with suspected sleep disorders, daily alcohol consumption, caffeine intake >400 mg/day, use of psychoactive substances, active smoking, or any known metabolic, cardiovascular, respiratory, or orthopedic conditions were excluded. One participant was subsequently excluded from analysis due to missing data. Descriptive characteristics are presented in Table 1. This study was approved by the Ethics Committee of UZ Leuven (S67790) and conducted in accordance with the Declaration of Helsinki. All participants provided written informed consent prior to participation.

**TABLE 1:** Subject characteristics.

Subject characteristic	Male	Female
Sample size	10	11
Age (years)	22.1 ± 2.2	22.4 ± 2.2
Body mass (kg)	72.4 ± 6.7	59.6 ± 8.2
Body height (m)	1.80 ± 0.06	1.67 ± 0.07
Km/week running	36.7 ± 25.2	27.6 ± 6.1
Speed at 1st lactate threshold (km/h)	11.6 ± 2.1	10.1 ± 1.2
Speed at 2nd lactate threshold (km/h)	14.7 ± 0.9	12.6 ± 0.8
Self-reported habitual sleep duration (h)	8.0 ± 0.9	8.3 ± 0.7

### 2.2 Study design

The full study design and experimental protocol are described in detail elsewhere (Seynaeve et al., 2026). Briefly, an incremental treadmill test and a sleep-lab habituation visit were both completed 1 week before the first experimental session. Then, participants completed two experimental sessions in a randomized, counterbalanced crossover design: one following a night of normal sleep (control condition, CON) and one following a night of total sleep deprivation (SDEP), separated by a one-week washout period (Figure 1). In the CON condition, participants slept in the laboratory from 11:00 pm to 07:00 am under full polysomnographic monitoring. In the SDEP condition, participants remained awake throughout the night under continuous supervision. The running protocol commenced at 07:30 am following CON and at 06:30 am following SDEP; this earlier start for SDEP reflected practical necessity, as participants in this condition had already been awake throughout the night, whereas participants in the CON condition required additional time after waking for sleep inertia to dissipate before testing began.

**FIGURE 1:**
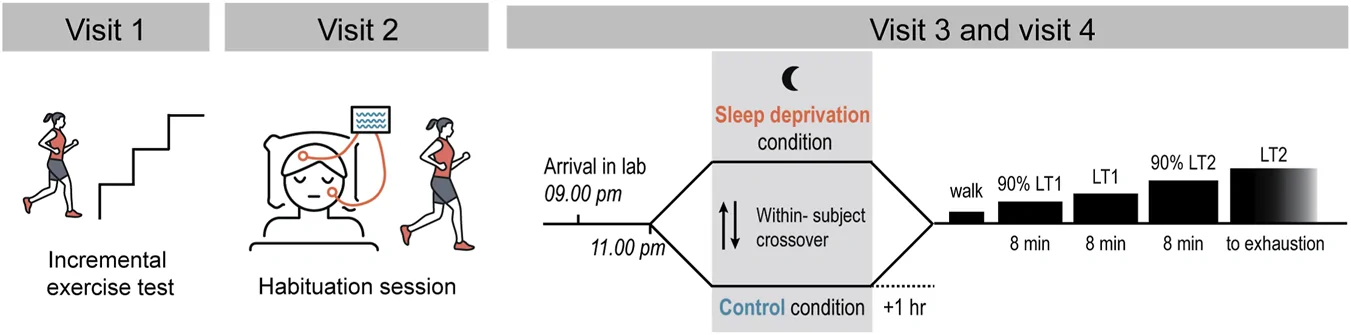
Schematic overview of the study visits. During visit 1, participants completed an incremental exercise test to determine individual lactate thresholds. Visit 2 served as a habituation session, familiarizing participants with the sleep laboratory and the running protocol. Both visits took place 1 week before the first experimental session (visit 3). Visits 3 and 4 were completed in a counterbalanced, randomized order, consisting of one sleep deprivation night and one control night. The morning following each experimental night, participants performed three submaximal running intervals of 8 min at increasing intensities, followed by a single interval to volitional exhaustion at their second lactate threshold. LT1, first lactate threshold; LT2, second lactate threshold. Adapted with permission from Wolters Kluwer Health, LLC: Seynaeve M, Vanwanseele B, de Beukelaar T. One night of acute sleep deprivation alters running biomechanics and running economy in recreational runners. Medicine & Science in Sports & Exercise (official journal of the American College of Sports Medicine). Published ahead of print 9 July 2026. doi:10.1249/MSS.0000000000004081. The Creative Commons license does not apply to this content. Use of the material in any format is prohibited without written permission from the publisher, Wolters Kluwer Health, LLC. Please contact permissions@lww.com for further information.

The running protocol consisted of three 8-min intervals at 90% of LT1, LT1, and 90% of LT2, followed by a fourth interval at LT2 speed continued until volitional exhaustion. LT1 (the first lactate threshold, or aerobic threshold) broadly corresponds to a low-to-moderate training intensity (comparable to an “easy/steady” heart-rate zone at which conversation remains possible), whereas LT2 (the second lactate threshold, or maximal lactate steady state) corresponds to a harder, near-race-pace intensity at a substantially higher heart rate. All running trials were performed on an instrumented treadmill (M-Gait, Motek, Amsterdam, the Netherlands). Running intensities were individually determined based on lactate thresholds established during a prior incremental treadmill test (4-min stages, starting at 5.4 km/h and increasing by 1.8 km/h per stage until volitional exhaustion).

### 2.3 Instrumentation

Two motion capture systems were used simultaneously to record running biomechanics during each session. Laboratory-based kinematics were recorded using a 13-camera infrared motion capture system (Vicon, Oxford Metrics, Oxford, UK) sampling at 250 Hz, with 28 reflective markers. Concurrently, trunk accelerometry was recorded using the Runeasi system, a commercially available wearable sensor (Movesense, Vantaa, Finland) with integrated cloud-based processing platform (Runeasi.ai, Tienen, Belgium), sampling at 200 Hz. The sensor was mounted at the lower back between L5 and S2 using a firm elastic belt approximating the center of mass. The underlying trunk-accelerometer approach has been developed and evaluated across a series of peer-reviewed studies by the same research group. Accelerometer-derived loading estimates have been compared against ground-reaction-force and GPS-based measures (Vanwanseele et al., 2020), and the approach has been applied to detect effects of running surface (Schütte et al., 2016), fatigue-related and injury-related changes in loading and dynamic stability (Schütte et al., 2015; Schütte et al., 2018), and prospective lower-leg overuse injury risk (Pla et al., 2021). Both systems recorded simultaneously throughout all running intervals, allowing direct comparison of their discriminative capacity under identical experimental conditions.

### 2.4 Feature extraction

For the lab-based motion capture system, biomechanical features were extracted from the spatiotemporal, kinematic, kinetic and joint power variables described in detail elsewhere (Seynaeve et al., 2026). Variables were computed over strides collected between minutes 5 and 7 of the first three intervals and minutes 1 to 3 of the final interval. The minute 5–7 window was used for the first three (fixed-duration) intervals to ensure steady-state running was reached before feature extraction. The final interval was sampled earlier (minutes 1–3) because it continued until volitional exhaustion and therefore had a variable total duration across participants; an early window ensured comparable, non-fatigue-confounded data availability across all participants. Only features normalized to body weight were used in the analysis. A total of 83 features were included in the final dataset.

For the wearable sensor, raw acceleration signals were automatically processed by the Runeasi platform into 21 running metrics computed in 5-s epochs. Epochs containing data corresponding to minutes 5 to 7 of the first three intervals and minutes 1 to 3 of the final interval were summarized by computing the mean, standard deviation, and left-right asymmetry of each metric, yielding 36 features per interval per participant. Left–right asymmetry was calculated as the absolute, non-directional percentage difference between limbs, ensuring that only the magnitude of inter-limb differences was retained. The extracted metrics included spatiotemporal characteristics (cadence, flight ratio, flight time, ground contact time), impact-related variables (impact magnitude, impact duration, peak loading rate, cumulative impact), trunk movement qualities (dynamic stability, smoothness), and anteroposterior/vertical ground reaction forces (braking, braking impulse, propulsive impulse, vertical impulse). A full description of each metric and its computation is provided in Supplementary Material S1.

### 2.5 Classification task

Two complementary classification tasks were evaluated. The first was a within-subject task, in which paired data from CON and SDEP sessions of a single participant were presented to the classifier. Then, the classifier was required to determine which session corresponded to each condition. This formulation explicitly removes inter-subject variability and isolates the intra-individual gait change due to sleep deprivation. Although CON − SDEP and SDEP − CON difference vectors are exact sign-reversals of one another, this does not trivially inflate classification accuracy: because each subject contributes exactly one example of each class, the task remains a balanced binary discrimination with a 50% chance baseline, and successful classification still requires the model to detect a genuine, consistent directional shift in the underlying features rather than exploiting class imbalance or other artifacts.

The second was a between-subject task in which raw session measurements were classified as CON or SDEP without pairing, requiring the classifier to generalize across the natural variation in running gait between individuals. The between-subject task is more challenging and more generally applicable in a real-world setting, where a reference recording under rested conditions may not be available. Both approaches were applied independently to features derived from the lab system and from the wearable sensor, yielding four classification pipelines in total. In all cases, a single group-level model was trained across participants rather than subject-specific models.

### 2.6 Classification models

Five supervised classification algorithms were evaluated: Logistic Regression, Decision Tree, Random Forest, k-Nearest Neighbors (k-NN), and Extreme Gradient Boosting (XGBoost). These algorithms were chosen to span complementary modeling paradigms commonly used in gait- and fatigue-detection research (Buckley et al., 2017; Op De Beéck et al., 2018; Dimmick et al., 2023). Logistic Regression is a linear model that estimates class log-odds from a weighted combination of features and yields directly interpretable coefficients (Hosmer et al., 2013). Decision Tree recursively partitions the feature space into simple, interpretable decision rules (Blockeel et al., 2023). Random Forest is a bagged ensemble of decision trees that reduces variance and improves robustness relative to a single tree (Breiman, 2001). k-NN is a non-parametric, distance-based method that classifies observations according to the majority label among their nearest neighbors, capturing local, potentially non-linear structure (Kramer, 2013). XGBoost is a gradient-boosted ensemble of decision trees that sequentially corrects the errors of preceding trees and typically performs well on structured, tabular data (Chen et al., 2015). Together, these models allowed comparison of linear, single-tree, bagged-ensemble, distance-based, and boosted-ensemble approaches within the same evaluation framework.

Each model was subjected to systematic hyperparameter optimization using a nested cross-validation scheme, with the number of retained features treated as a tunable hyperparameter and optimized jointly with the model-specific parameters. Analyses were conducted separately for the two motion capture systems (lab and wearable) and for both classification tasks (within- and between-subject). All analyses were implemented in Python using *scikit-learn*, *XGBoost*, and *statsmodels*. A fixed random seed (42) was used throughout for reproducibility.

### 2.7 Data preparation

#### 2.7.1 Within-subject task

For each motion capture system, subject and running speed, biomechanical features from the CON and SDEP sessions were subtracted to produce two signed difference vectors: CON - SDEP and SDEP - CON. This way, each subject contributed two data points per speed condition. This formulation eliminates between-subject baseline differences and constrains the problem to detecting the directional shift in gait attributable to sleep deprivation.

#### 2.7.2 Between-subject task

The same subjects and speed conditions were used, but the raw session measurements were retained without pairing. Each observation was labeled as CON or SDEP, and the classifier was required to generalize across subjects without access to a paired reference.

For both tasks, features were organized into a feature matrix X, a binary outcome vector y, and a group vector indicating subject identity. Running speed was encoded as a categorical variable with four levels (1–4), and used as a covariate in the ANOVA-based feature selection step.

### 2.8 Processing pipeline

For each cross-validation fold, a three-stage pipeline was applied strictly within the training partition to prevent data leakage. *First*, all features were standardized to zero mean and unit variance using a standard scaler fitted on training data only. *Second*, a feature selector based on an analysis of variance (ANOVA) identified the features most strongly associated with condition type. Specifically, for each candidate feature, a two-way ANOVA (Type II) was fitted with condition and running speed as factors, including their interaction term. Features were ranked by the *p*-value of the condition main effect and the top *k* retained, where *k* was treated as a hyperparameter. This univariate ANOVA-based filter was applied identically to all five algorithms, including the tree-based models, so that the feature selection step remained the same and directly comparable across classifiers. *Third*, the selected classifier was trained on the standardized, reduced feature set.

### 2.9 Nested cross-validation and hyperparameter optimization

A nested cross-validation framework was used to provide an unbiased estimate of generalization performance. The outer loop used 10-fold group cross-validation (GroupKFold) stratified by subject identity, ensuring no subject appeared in both training and test partitions within a fold. For each outer training set, an inner GroupKFold grid search (up to five folds, capped by available subjects) exhaustively evaluated all hyperparameter combinations using accuracy as the criterion, and the best configuration was refitted on the full outer training set before evaluation on the held-out fold.

Hyperparameter search spaces were as follows. Logistic Regression: regularization strength *C* ∈ {0.001, 0.01, 0.1, 1, 10, 100}, *liblinear* solver. Decision Tree: maximum depth ∈ {2, 3, 4, unlimited}, minimum samples to split ∈ {2, 4, 6}. Random Forest: number of trees ∈ {50, 100, 200}, maximum depth ∈ {2, 3, unlimited}. k-NN: neighbors ∈ {3, 5, 7}, weighting ∈ {uniform, distance}. XGBoost: boosting rounds ∈ {5, 10, 50, 100}, maximum depth ∈ {2, 3, unlimited}, learning rate ∈ {0.01, 0.1, 0.2}. For all models, the number of ANOVA-selected features *k* was searched over {5, 10, 20, all}.

Classification accuracy was recorded per outer fold and summarized as mean ± standard deviation across ten folds. A chance-level baseline of 50% applies to both tasks given the balanced binary design. Sensitivity, specificity, and ROC-AUC were likewise computed on each of the ten outer folds, with sleep deprivation (SDEP) treated as the positive class: sensitivity is the recall for SDEP (true positive rate) and specificity is the recall for CON (true negative rate). ROC-AUC was computed from the model’s predicted probability of the SDEP class.

### 2.10 Permutation testing and confidence intervals

To assess whether classification accuracy exceeded chance level, label-permutation testing was performed separately for each model, motion capture system, and classification task (i.e., separately for all 5×2×2=20 model/system/task combinations). For each of 1,000 permutations per combination, condition labels (CON/SDEP) were swapped at the subject level: for every subject, a single coin flip determined whether *all* of that subject’s observations (across all speeds and both sessions) had their condition label swapped, or none of them did. This preserves the within-subject structure of the data (repeated measurements from the same person under the same condition stay internally consistent) while breaking the condition-related signal the test is designed to probe. For computational tractability, hyperparameters were fixed to the per-fold-optimal values identified during the original nested cross-validation on the real (unpermuted) data (i.e., the configuration selected by the inner grid search for each outer fold), rather than re-optimized within each permutation; the ANOVA feature-selection step was nonetheless refitted on the permuted training labels within each fold, to prevent leakage of real-label feature rankings into the null distribution. The outer 10-fold GroupKFold cross-validation was otherwise repeated in full for each permutation, using the same fold boundaries as the real-data analysis, so that the null distribution reflects the same evaluation procedure as the reported accuracies. The permutation *p*-value was computed as p=(#{permuted mean accuracies≥observed mean accuracy}+1)/(N+1) , with N=1,000 .

Ninety-five percent confidence intervals for accuracy were computed from the ten outer-fold scores using the t -distribution: $\text{mean}\pm t_{0.975,\;df=9}\times \text{SE}$ , where SE is the standard error of the fold scores. Because outer folds are not fully independent (training partitions overlap across folds), this interval is slightly anti-conservative; it follows standard convention for reporting cross-validation results in the machine-learning literature.

### 2.11 Feature importance analysis

To identify which gait features drove classification, each model was refitted on the full dataset and feature importances were extracted: absolute logistic regression coefficients for Logistic Regression, and Gini-based impurity reduction for Decision Tree, Random Forest, and XGBoost. k-NN does not yield intrinsic feature importances and was excluded from this analysis. Feature importances were computed separately for each sensor system and classification task.

## 3 Results

### 3.1 Within-subject classification

For the within-subject task, we classified the direction of the within-individual gait difference (CON - SDEP vs. SDEP - CON). Label-permutation testing and confidence intervals (Figure 2; Table 2) showed that classification accuracy was significantly above chance (p<0.05) for eight of the ten model/system combinations; the exceptions were Logistic Regression ( p = 0.089) and k-Nearest Neighbors ( p = 0.163) on the lab-based dataset, which did not reach significance despite numerically exceeding 50%. This indicates that sleep deprivation produces a systematic and directionally consistent change in running gait within individuals that is detectable by machine learning classifiers for most, though not all, models and systems.

**FIGURE 2:**
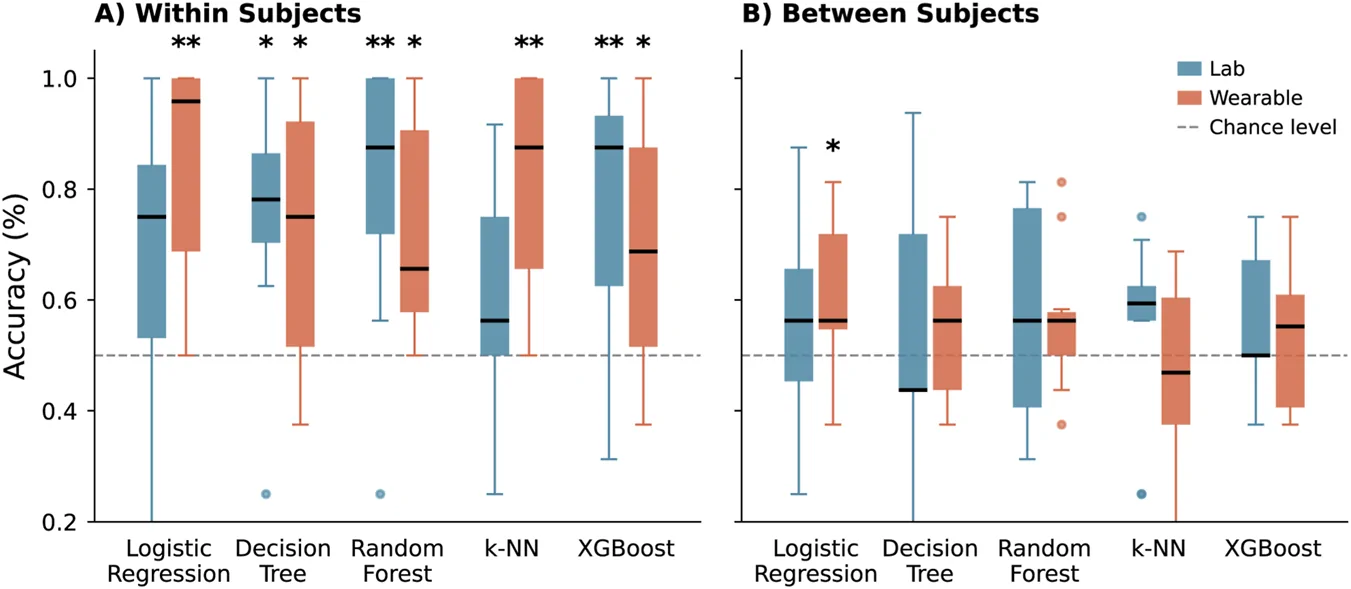
Classification accuracy of machine learning models for the within-subject **(A)** and between-subject task **(B)**. Box plots showing cross-validated accuracy scores for five machine learning models evaluated on the lab-based (blue) and wearable (red) datasets. Each box represents the distribution of accuracy scores across cross-validation folds, with the horizontal line indicating the median, the box boundaries showing the interquartile range, and individual points representing outliers. The dashed horizontal line marks chance level (0.5). Both datasets were evaluated on identical model architectures with hyperparameter optimization performed via grid search. Asterisks indicate accuracy significantly above chance level based on label-permutation testing ( N = 1,000 permutations per model/system/task; * p<0.05 , ** p<0.01 ); the absence of an asterisk indicates no significant difference from chance. Classification accuracy was significantly above chance for most models and both motion capture systems on the within-subject task, but for only one model/system combination (wearable Logistic Regression) on the between-subject task (see Tables 2, 3 for exact accuracies, confidence intervals, and p -values).

**TABLE 2:** Within-subject classification performance across models and motion capture systems, including permutation-based significance testing, confidence intervals, ROC-AUC, sensitivity, and specificity.

Model	Accuracy (%)	95% CI	*p*	AUC (%)	Sens. (%)	Spec. (%)
Lab-based motion capture						
Logistic regression	66.2 ± 25.0	[47.4, 85.1]	0.089	73.1 ± 27.8	66.2 ± 25.0	66.2 ± 25.0
Decision tree	74.6 ± 19.3	[60.0, 89.2]	0.012*	73.2 ± 18.2	71.7 ± 28.4	77.5 ± 20.8
Random forest	**80.6** ± **23.3**	[63.1, 98.2]	0.005*	83.9 ± 24.3	79.6 ± 23.1	81.7 ± 24.0
k-Nearest neighbors	60.4 ± 20.6	[44.9, 76.0]	0.163	64.4 ± 26.3	60.4 ± 20.6	60.4 ± 20.6
XGBoost	77.9 ± 21.2	[61.9, 93.9]	0.009*	83.3 ± 22.8	76.7 ± 22.2	79.2 ± 20.4
Wearable sensor						
Logistic regression	**84.2** ± **20.3**	[68.9, 99.5]	0.003*	84.9 ± 24.7	84.2 ± 20.3	84.2 ± 20.3
Decision tree	72.1 ± 21.7	[55.7, 88.5]	0.016*	69.4 ± 21.9	73.8 ± 22.7	70.4 ± 21.4
Random forest	72.1 ± 18.8	[57.9, 86.3]	0.022*	78.7 ± 18.7	72.9 ± 17.9	71.2 ± 20.2
k-Nearest neighbors	82.5 ± 17.9	[69.0, 96.0]	0.001*	86.6 ± 15.7	82.5 ± 17.9	82.5 ± 17.9
XGBoost	69.8 ± 20.1	[54.7, 84.9]	0.041*	78.4 ± 20.4	71.7 ± 19.8	67.9 ± 20.8

^*^ p<0.05 (label-permutation test, N = 1,000 permutations). Bold indicates the highest accuracy per motion capture device. Because both classes are perfectly balanced in every outer-fold test set for this task, accuracy is mathematically the mean of sensitivity and specificity for every model.

For the lab-based motion capture dataset, Random Forest led with a mean accuracy of 80.6% ± 23.3% ( p = 0.005), followed by XGBoost (77.9% ± 21.2%, p = 0.009) and Decision Tree (74.6% ± 19.3%, p = 0.012). Logistic Regression (66.2% ± 25.0%, p = 0.089) and k-NN (60.4% ± 20.6%, p = 0.163) trailed the other models and did not reach statistical significance. This pattern suggests that the richer, higher-dimensional motion capture feature space contains non-linear structure better exploited by tree-based and ensemble methods, while the linear and distance-based models struggled to capture this structure.

For the wearable sensor dataset, Logistic Regression achieved the highest mean accuracy (84.2% ± 20.3%, p = 0.003), closely followed by k-NN (82.5% ± 17.9%, p = 0.001). Random Forest (72.1% ± 18.8%, p = 0.022) and Decision Tree (72.1% ± 21.7%, p = 0.016) performed at an intermediate level, and XGBoost (69.8% ± 20.1%, p = 0.041) was lowest, though still statistically significant. The strong performance of the linear model again suggests that the within-subject gait difference captured by the wearable sensor is relatively low-dimensional and linearly separable, though the competitive performance of k-NN indicates a degree of local structure in the feature space as well.

Fold-level variability was substantial across both datasets. The best-performing model for the wearable dataset (Logistic Regression) showed a standard deviation of 20.3%, while the best-performing model for the lab-based dataset (Random Forest) showed a standard deviation of 23.3%, with some folds yielding perfect classification and others falling near or below chance. This indicates meaningful between-subject heterogeneity in the magnitude, though not the direction, of the gait response to sleep deprivation.

### 3.2 Between-subject classification

For the between-subject task, we predicted session type (CON or SDEP) from raw gait measurements without paired reference data, again evaluated using label-permutation testing and confidence intervals (Figure 2; Table 3). This task proved substantially more difficult: only one of the ten model/system combinations, Logistic Regression on the wearable dataset ( p = 0.033), reached statistical significance; all other models performed close to or only modestly above the 50% chance level and did not differ significantly from chance. This sharp decline relative to the within-subject task suggests that inter-subject variability in running gait may outweigh the sleep-deprivation signal when individuals are treated independently.

**TABLE 3:** Between-subject classification performance across models and motion capture systems, including permutation-based significance testing, confidence intervals, ROC-AUC, sensitivity, and specificity.

Model	Accuracy (%)	95% CI	*p*	AUC (%)	Sens. (%)	Spec. (%)
Lab-based motion capture						
Logistic regression	55.4 ± 18.3	[41.6, 69.2]	0.218	64.0 ± 15.2	47.1 ± 25.7	63.7 ± 37.7
Decision tree	53.1 ± 22.2	[36.4, 69.9]	0.339	53.2 ± 24.1	46.2 ± 30.6	60.0 ± 30.0
Random forest	**57.3** ± **18.9**	[43.1, 71.5]	0.158	63.9 ± 26.9	54.6 ± 28.7	60.0 ± 38.2
k-Nearest neighbors	55.2 ± 16.2	[43.0, 67.4]	0.196	62.1 ± 20.1	52.5 ± 20.8	57.9 ± 29.8
XGBoost	56.9 ± 12.0	[47.8, 65.9]	0.164	61.9 ± 22.1	57.5 ± 34.6	56.2 ± 34.1
Wearable sensor						
Logistic regression	**60.4** ± **14.1**	[49.8, 71.0]	0.033*	72.8 ± 18.4	64.6 ± 18.5	56.2 ± 34.1
Decision tree	54.8 ± 11.6	[46.1, 63.5]	0.156	55.8 ± 10.3	55.0 ± 22.5	54.6 ± 21.7
Random forest	56.5 ± 12.5	[47.0, 65.9]	0.109	61.7 ± 15.8	57.9 ± 32.3	55.0 ± 27.5
k-Nearest neighbors	46.7 ± 16.5	[34.2, 59.1]	0.748	46.2 ± 19.7	45.0 ± 19.5	48.3 ± 26.9
XGBoost	52.9 ± 11.9	[43.9, 61.9]	0.285	61.1 ± 16.6	55.0 ± 27.5	50.8 ± 26.3

* p<0.05 (label-permutation test, N = 1,000 permutations). Bold indicates the highest accuracy per system.

For the lab-based dataset, performance was constrained across all models, and none reached statistical significance. Random Forest performed marginally best (57.3% ± 18.9%, p = 0.158), followed by XGBoost (56.9% ± 12.0%, p = 0.164), Logistic Regression (55.4% ± 18.3%, p = 0.218), k-NN (55.2% ± 16.2%, p = 0.196), and Decision Tree (53.1% ± 22.2%, p = 0.339).

For the wearable dataset, Logistic Regression again achieved the highest accuracy (60.4% ± 14.1%), and was the only model to reach statistical significance ( p = 0.033). Random Forest (56.5% ± 12.5%, p = 0.109) and Decision Tree (54.8% ± 11.6%, p = 0.156) showed modest, non-significant improvements over chance, while XGBoost (52.9% ± 11.9%, p = 0.285) and k-NN (46.7% ± 16.5%, p = 0.748) performed at or below chance level on average. Nevertheless, no model achieved classification accuracy that would be interpretable as reliable detection of sleep deprivation at the group level without individual reference data, with only the wearable Logistic Regression model reaching statistical significance, and even there accuracy remained only modestly above chance level.

### 3.3 Feature importance

Feature importances were computed on the full dataset for the within-subject task, providing insight into which gait characteristics are most sensitive to sleep deprivation at the individual level (Figure 3). These values were obtained by refitting each model on the full dataset rather than aggregating importance estimates across held-out cross-validation folds; they should therefore be interpreted as exploratory rather than as validated, robust biomarkers of sleep-deprivation sensitivity.

**FIGURE 3:**
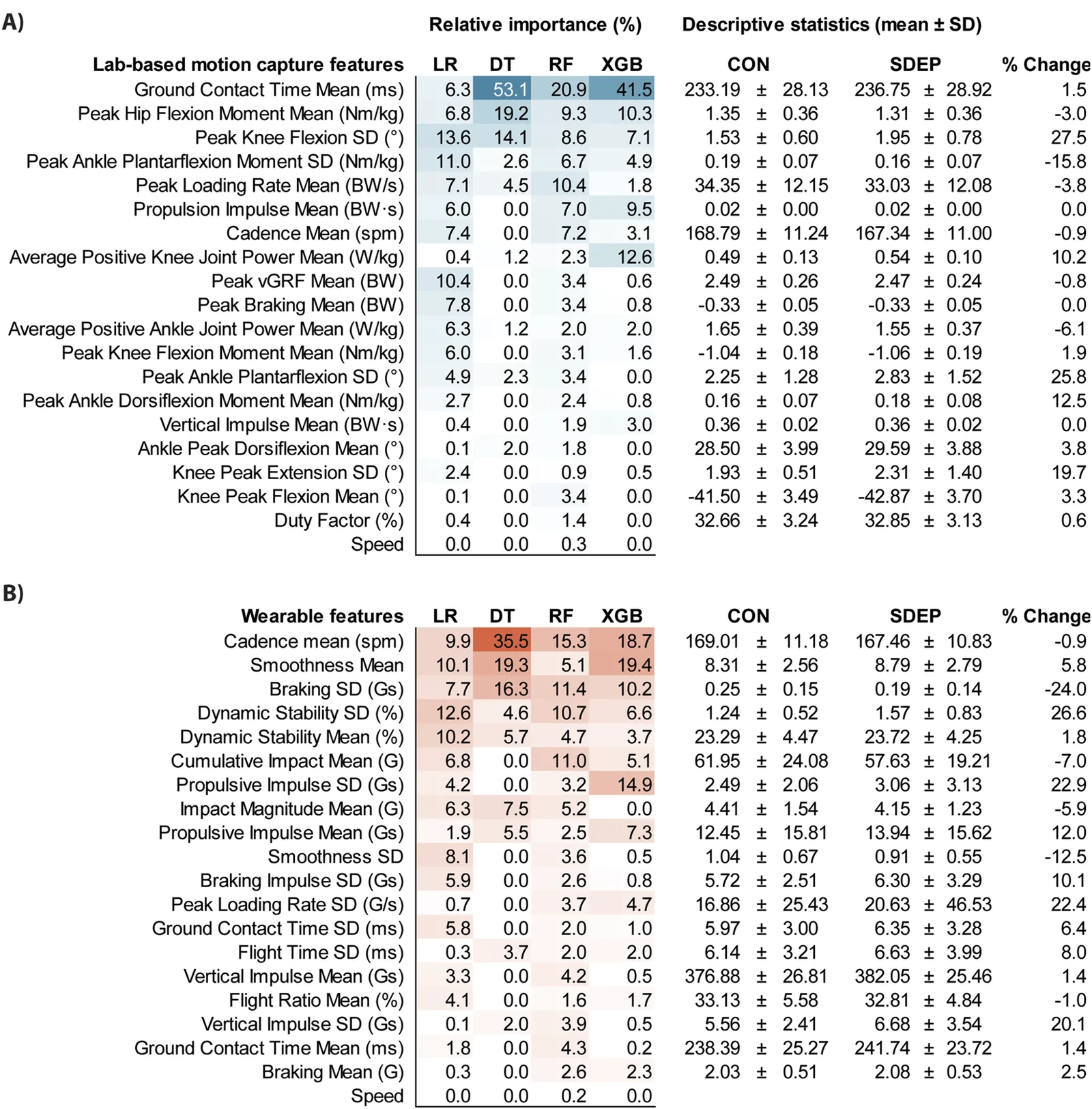
Feature importance heatmap across four classifier models for the within-subject task. Normalized feature importance (0–100) of **(A)** lab-based motion capture features (blue), and **(B)** wearable-derived features (red). Features are ranked by mean importance; darker shading = higher importance. Normalization was applied within each model. Across both systems, variability-based features (SD metrics) were consistently among the most important discriminators, alongside spatiotemporal features such as ground contact time and cadence. Importance was highly concentrated in a single feature for the decision tree, whereas Random Forest and XGBoost showed more distributed profiles across both the lab-based and wearable systems. For each feature, the figure additionally reports descriptive statistics (CON and SDEP mean ± SD, and percent change) alongside the importance rankings, giving the direction and magnitude of the underlying condition difference for every feature shown. LR, Logistic Regression; DT, Decision Tree; RF, Random Forest; XGB, XGBoost.

#### 3.3.1 Lab-based dataset

Ground contact time was the dominant feature for tree-based models, ranking first for Decision Tree (53.1%), Random Forest (20.9%), and XGBoost (41.5%), though it contributed less distinctively in Logistic Regression, where importance was more evenly distributed across features. Peak hip flexion moment was the second most important feature overall, contributing consistently across all four models (6.8%, 19.2%, 9.3%, 10.3%). Peak knee flexion variability also appeared among the top features across all models (13.6%, 14.1%, 8.6%, 7.1%), followed by peak ankle plantarflexion moment variability and peak loading rate as moderately important contributors. Overall, the pattern points to sleep deprivation altering ground contact mechanics and lower-limb loading characteristics in a consistent within-individual direction, with kinetic and spatiotemporal variables jointly driving classification across the full-body motion capture feature space.

#### 3.3.2 Wearable dataset

Cadence was the single most consistently important feature across models, ranking first for Logistic Regression (9.9%), Decision Tree (35.5%), and Random Forest (15.3%), and second for XGBoost (18.7%). Smoothness mean was similarly prominent, ranking first for XGBoost (19.4%) and second for Decision Tree (19.3%), and contributing meaningfully in Logistic Regression (10.1%). Braking SD emerged as a consistently important contributor across all four models (7.7%, 16.3%, 11.4%, 10.2%), as did Dynamic Stability SD (12.6%, 4.6%, 10.7%, 6.6%). Beyond these leading features, cumulative impact mean and propulsive impulse variability appeared as moderately important for Logistic Regression, Random Forest and XGBoost. The prominence of variability features alongside mean-level variables suggests that sleep deprivation affects not only the central tendency of key gait parameters but also the consistency of movement mechanics within individuals.

## 4 Discussion

This study examined whether total sleep deprivation produces detectable changes in running gait biomechanics. Analyses were performed using two complementary motion capture systems - a laboratory-based full-body system (Vicon) and a field-deployable trunk-mounted wearable (Runeasi) - and two classification tasks that differed depending on the availability of an individual reference condition. These analyses resulted in three primary findings. *First*, within-subject classification was above chance (label-permutation testing, p<0.05 ) for most models and both motion capture systems, suggesting that sleep deprivation produces a systematic and individually consistent biomechanical signature in running gait. *Second*, between-subject classification failed across nearly all models and both motion capture systems, suggesting that inter-individual variability in running gait may overwhelm the sleep-deprivation signal when no reference condition is available. *Third*, despite their fundamental differences in measurement scope, both systems converged on temporal, loading-related, and variability-based features as the most discriminative signals of sleep deprivation. These findings partially support our hypotheses: sleep deprivation was detectable above chance for the within-subject task, and within-subject classification outperformed between-subject classification as anticipated. However, contrary to our expectation that the lab-based motion capture would outperform the wearable by virtue of its richer feature space, both systems achieved comparable within-subject classification accuracy, and neither system supported meaningful between-subject detection.

### 4.1 Within-subject vs. between-subject classification

Within-subject classification accuracy was significantly above chance level (label-permutation testing, p<0.05 ) for eight of the ten model/system combinations, and both measurement modalities were represented among the significant models; the two exceptions (lab-based Logistic Regression and k-Nearest Neighbors) were the weakest-performing models on the lab-based dataset. This provides evidence that total sleep deprivation alters running gait in a sufficiently systematic and repeatable manner within individuals to support machine learning classification. These findings extend recent laboratory-based work demonstrating meaningful biomechanical changes following a single night of sleep deprivation by showing that such alterations are sufficiently structured to permit condition discrimination at the individual level (Seynaeve et al., 2026). However, fold-level variability (i.e., the spread in classification accuracy observed across the ten outer cross-validation folds, each corresponding to a different held-out subset of participants) was substantial across both systems, with individual folds ranging from near-perfect classification to near-chance performance. With only 21 participants, each outer fold held out data from just two to three individuals at a time, so fold-level accuracy is particularly sensitive to the specific characteristics of whichever participants happened to be excluded; this sample-size constraint likely amplifies the observed variability beyond what a larger cohort would produce, and the point estimates of accuracy reported here should therefore be interpreted alongside their considerable uncertainty rather than as precise, stable values. Additionally, heterogeneity in the magnitude of the gait response to sleep deprivation across individuals may also have contributed. Such variability is nonetheless consistent with well-documented inter-individual differences in vulnerability to sleep deprivation (Dongen et al., 2004).

In contrast to within-subject classification, between-subject classification failed to produce meaningful discrimination in either modality, with most models performing within a few percentage points of the 50% chance baseline. As documented extensively in the exercise-induced fatigue detection literature, inter-individual differences in gait patterns often exceed the magnitude of condition-induced effects (Chalitsios et al., 2024). The present findings suggest that this challenge extends to the sleep deprivation context. More broadly, these results support the subject-specific, baseline-normalized approach advocated in the fatigue literature (Op De Beéck et al., 2018), indicating that the absence of an individual reference measurement substantially limits the reliable detection of sleep-deprivation-related gait changes. In practical terms, a single gait recording obtained without knowledge of an individual’s rested running pattern appears insufficient to reliably infer sleep deprivation status.

### 4.2 Wearable sensors vs. 3D motion capture

Contrary to our hypothesis that 3D motion capture would outperform a wearable system, both systems achieved comparable within-subject classification accuracy, and neither supported meaningful between-subject detection. However, the optimal model family differed notably between the two systems. Using wearable sensor features, linear and distance-based models (Logistic Regression and k-NN) outperformed tree-based ensemble methods (Random Forest and XGBoost), whereas the reverse pattern was observed for the lab-based motion capture features.

The interpretation of this dissociation warrants caution, as model performance alone cannot establish the underlying geometry of a feature space, and multiple explanations may contribute. One plausible contributor is the difference in feature dimensionality between the two candidate pools. Large-scale benchmarking across 243 datasets has shown that the number of features is significantly correlated with the performance advantage of Random Forest over Logistic Regression, with the advantage shifting from negative to positive as dimensionality increases, while sample size alone was not a significant predictor of this gap (Couronné et al., 2018). The lab-based motion capture system drew from a substantially larger and more heterogeneous candidate pool of 83 features spanning multiple biomechanical domains, compared to 36 features for the wearable system. If the effective dimensionality and structural complexity of the retained feature sets differed accordingly, this could partly explain why ensemble methods outperformed linear models for the lab-based system but not for the wearable system.

A further, non-mutually-exclusive explanation concerns feature intercorrelation introduced by the selection procedure. Because ANOVA-based univariate feature selection ranks features by their individual association with the outcome without accounting for redundancy among predictors (Michalak and Kwasnicka, 2010; Mwadulo, 2016), features selected from the larger, more heterogeneous lab-based pool may carry greater residual intercorrelation. Whether this differentially affected the two model families is difficult to establish without formal analysis of inter-feature correlations in the retained subsets, and this explanation should be treated as a plausible hypothesis rather than a definitive account of the observed performance differences.

### 4.3 Feature importance

Feature importances were computed on the full dataset for the within-subject task to identify which features contributed most strongly to discrimination between rested and sleep-deprived conditions at the individual level. Despite substantial differences in feature representation between the two systems, comparison of importance patterns revealed convergence across three recurring themes, each described below together with the direction of the underlying condition difference; full descriptive statistics (CON and SDEP mean ± SD, percent change) for every feature are reported directly in Figure 3. The most pronounced concerned temporal structure: cadence was the dominant wearable feature across models, while ground contact time emerged as the dominant laboratory-based feature. These variables represent closely related components of stride-cycle timing. Therefore, their co-emergence as highly discriminative features across independent motion capture systems suggests that alterations in the temporal organization of running were among the most consistently discriminative signals of sleep deprivation. In direction, both features shifted toward a slower stride pattern in SDEP: cadence was slightly lower (−0.9%) and ground contact time was longer (+1.5%) than in CON, consistent with a more prolonged stance phase under sleep deprivation.

A second recurring theme involved loading-related information. Impact-related wearable features, including cumulative impact and impact magnitude, and laboratory-derived kinetic variables, including peak loading rate and peak braking force, all contributed meaningfully to classification. While trunk accelerometry provides an indirect representation of impact transmission, laboratory-based kinetics directly characterize ground reaction force mechanics. Therefore, these variables reflect related but not equivalent aspects of impact behavior. Nevertheless, their parallel importance suggests that loading-related information contributed substantially to discrimination across measurement contexts. These loading-related features consistently decreased in SDEP relative to CON: cumulative impact (−7.0%) and impact magnitude (−5.9%) on the wearable side, and peak loading rate (−3.8%) on the lab side, pointing toward an overall reduction in loading exposure under sleep deprivation.

A third shared pattern was the consistent contribution of variability-related features alongside mean-level variables in both systems. This indicates that stride-to-stride variability in key gait characteristics contributed meaningfully to discrimination between rested and sleep-deprived running states. Importantly, however, importance rankings alone cannot establish the direction of these changes; variability-related features may contribute strongly to classification regardless of whether variability increased, decreased, or shifted in a more complex multivariate manner. In the present sample, this variability increased for some features (peak-knee-flexion variability, +27.5%; dynamic-stability variability, +26.6%) but decreased for others (peak ankle plantarflexion moment variability, −15.8%; braking variability, −24.0%), indicating a mixed rather than uniform shift in movement variability under sleep deprivation.

The two systems diverged in ways that reflect their different measurement scope. Wearable importance was distributed more diffusely across trunk-derived variables including dynamic stability, smoothness, and multiple variability metrics, whereas laboratory-based importance concentrated on specific joint-level kinetic and kinematic variables, reflecting the system’s capacity to resolve mechanical detail inaccessible to a single trunk-mounted sensor. This suggests the two modalities may provide different but partially overlapping perspectives on the gait response to sleep deprivation, with the wearable capturing global coordination and trunk movement organization and the laboratory system isolating joint-specific adaptations.

The patterns described above are consistent with several hypothesized neuromuscular consequences of sleep deprivation, and find support in the descriptive data. First, impaired feedforward control may play a central role. Leg stiffness at ground contact depends in part on anticipatory muscle preactivation, governed by feedforward mechanisms that allow the nervous system to predict upcoming mechanical demands and adjust muscle activity accordingly (Müller et al., 2010; Müller et al., 2014; Khajooei et al., 2024). Sleep deprivation impairs higher-order sensorimotor and predictive control processes (Killgore, 2010; Umemura et al., 2021), which may reduce the precision with which muscle activation is timed and scaled before ground contact. This may leave the leg inadequately stiffened at touchdown, potentially contributing to the longer ground contact times, reduced cadence, and lower impact-related variables observed across both systems. A comparable reduction in leg stiffness and increase in knee flexion has been reported with exercise-induced fatigue, though that same evidence points to *increased*, rather than decreased, peak tibial impact loading (Zandbergen et al., 2023), suggesting that the loading reduction observed here may be a response more specific to centrally mediated, sleep-related fatigue. Second, the shift toward reduced loading may reflect a conservative protective strategy in which load reduction is prioritized over stiffness and propulsive efficiency when precise anticipatory control is compromised, a pattern previously proposed in the context of mountain ultra-marathon running (Vernillo et al., 2014; Degache et al., 2016). Third, the prominence of hip and knee kinetics alongside reduced ankle joint power in the lab-based dataset suggests a possible distal-to-proximal redistribution of joint work. During running, plantarflexors operate at a disproportionately high proportion of their maximal force and contribute a larger share of positive work than other muscle groups (Dorn et al., 2012; Kulmala et al., 2016), making them particularly susceptible to fatigue. A compensatory distal-to-proximal shift has been observed during prolonged running to exhaustion (Sanno et al., 2018; Willer et al., 2021), and it is plausible that centrally mediated fatigue under sleep loss may produce a similar pattern, though this remains speculative in the absence of direct electromyographic evidence. Finally, stride-to-stride inconsistency seemingly increased across the majority of kinematic and wearable-derived variability metrics. This is consistent with evidence that sleep deprivation impairs the regularity of continuous coordinated movement, likely through degraded sensorimotor feedback and executive control processes (Umemura et al., 2021; Umemura et al., 2022; Paillard, 2023).

However, it should be acknowledged that feature importance values reflect contribution to model discrimination rather than direct physiological sensitivity, and the following interpretations should be read as hypothesis-generating rather than conclusive. These mechanisms are not mutually exclusive, and because importance measures are operationalized differently across algorithms and are sensitive to feature covariance and model architecture, the patterns reported here should be interpreted as suggestive of recurring discriminative themes rather than definitive mechanistic conclusions. Similarly, the directional summaries reported above for individual features describe each feature’s own shift in isolation; some models, particularly the tree-based ensembles, may exploit combinations of features rather than any single feature’s shift alone, so these directions should be read as a summary of univariate trends rather than a complete account of the multivariate signal driving classification.

### 4.4 Limitations

Several limitations of the present study should be acknowledged. The sample size of 21 participants, while typical for laboratory-based gait studies and adequate for the within-subject design, limits statistical power and may contribute to the fold-level variability observed in cross-validation (Vabalas et al., 2019). Replication in larger samples is required to establish the robustness and generalizability of the findings.

The stability of selected features and model performance across resampling folds or participant subsets was not explicitly quantified, and the feature importance patterns reported should therefore be interpreted with caution. Feature importances were computed by refitting each model on the full dataset rather than aggregating importance estimates across held-out folds. This provides a descriptive overview of which features contribute most to in-sample classification, but the resulting estimates are exploratory in nature and not derived from independent out-of-sample evaluation. Moreover, importance measures are inherently model-dependent, differing across algorithms through coefficients, impurity-based measures, or gain metrics, and are sensitive to feature covariance, redundancy, and model structure (Nicodemus et al., 2010; Toloşi and Lengauer, 2011). As such, they should not be interpreted as direct indicators of physiological sensitivity. The same univariate ANOVA-based feature-selection filter was applied identically to all five models; while this ensured a directly comparable feature set across classifiers, it did not allow the tree-based algorithms (Decision Tree, Random Forest, XGBoost) to exploit correlated or non-linearly interacting predictors directly, a trade-off accepted for comparability rather than to maximize any individual model’s performance. Relatedly, we did not assess model calibration (i.e., whether predicted class probabilities reflect true outcome likelihoods) or evaluate performance on an independent external cohort. Both steps would be necessary before these models could be considered for applied or clinical use, hence they constitute priorities for future work.

The experimental protocol involved total sleep deprivation, which represents an extreme and relatively uncommon condition in real-world athletic settings. More ecologically prevalent forms of sleep restriction, such as partial sleep loss over multiple nights, may produce subtler or qualitatively different gait adaptations. It therefore remains uncertain to what extent the present findings generalize to more chronic or realistic sleep loss scenarios.

The wearable system (Runeasi) processes raw accelerometry through a proprietary algorithm, and the exact computational definitions of several derived metrics are not fully transparent. This limits mechanistic interpretability and reduces cross-platform reproducibility of specific features.

Finally, all running trials were performed on a treadmill at fixed speeds. Although this constrains ecological validity, it was a deliberate methodological choice: treadmill running allowed for precise speed control, which is essential given that speed directly influences cadence and other spatiotemporal parameters. It therefore remains unclear whether the observed gait changes would manifest similarly during overground running or at self-selected speeds, where pacing strategies may shift in response to fatigue and speed can no longer be held constant.

Taken together, these limitations highlight the need for cautious interpretation of the present findings and for future work to evaluate their robustness across larger samples, more ecologically valid sleep loss conditions, and more naturalistic running environments.

## 5 Conclusion

This study provides evidence that total sleep deprivation is associated with a detectable and individually consistent biomechanical signature in running gait, identifiable by both a laboratory motion capture system and a field-deployable trunk-mounted wearable. Within-subject classification performed significantly above chance for most models and both systems, whereas between-subject classification was significant for only one of ten model/system combinations, supporting the view that individual baseline data may be important for reliable gait-based fatigue detection. This conclusion mirrors findings from the exercise-induced fatigue literature and extends them to a sleep deprivation context. Contrary to our hypothesis, the lab-based motion capture system did not outperform the wearable device. Instead, both systems captured complementary rather than redundant aspects of the gait response. Together, these findings provide a biomechanical and methodological foundation for wearable-based fatigue monitoring in runners, and highlight longitudinal, individualized monitoring as a promising approach for detecting sleep-deprivation effects in real-world running contexts.

## Data Availability

The raw data supporting the conclusions of this article will be made available by the authors, without undue reservation.
